# Applications of Circular Dichroism for Structural Analysis of Gelatin and Antimicrobial Peptides

**DOI:** 10.3390/ijms13033229

**Published:** 2012-03-08

**Authors:** Ramamourthy Gopal, Jin Soon Park, Chang Ho Seo, Yoonkyung Park

**Affiliations:** 1 Research Center for Proteineous Materials, Chosun University, Gwangju 501-759, Korea; E-Mail: ramagopa@gmail.com; 2 Department of Biotechnology, Chosun University, Gwangju 501-759, Korea; E-Mail: jsmw2001@naver.com; 3 Department of Bioinformatics, Kongju National University, Kongju, South Korea; E-Mail: chseo@kongju.ac.kr

**Keywords:** circular dichroism, antimicrobial peptides, reduced glutathione, gelatin, sodium dodecyl sulfate, Tween 80, cell wall components, lipopolysaccharide

## Abstract

Circular dichroism (CD) is a useful technique for monitoring changes in the conformation of antimicrobial peptides or gelatin. In this study, interactions between cationic peptides and gelatin were observed without affecting the triple helical content of the gelatin, which was more strongly affected by anionic surfactant. The peptides did not adopt a secondary structure in the presence of aqueous solution or Tween 80, but a peptide secondary structure formed upon the addition of sodium dodecyl sulfate (SDS). The peptides bound to the phosphate group of lipopolysaccharide (LPS) and displayed an alpha-helical conformation while (KW)_4_ adopted a folded conformation. Further, the peptides did not specifically interact with the fungal cell wall components of mannan or laminarin. Tryptophan blue shift assay indicated that these peptides interacted with SDS, LPS, and gelatin but not with Tween 80, mannan, or laminarin. The peptides also displayed antibacterial activity against *P. aeruginosa* without cytotoxicity against HaCaT cells at MIC, except for HPA3NT3-analog peptide. In this study, we used a CD spectroscopic method to demonstrate the feasibility of peptide characterization in numerous environments. The CD method can thus be used as a screening method of gelatin-peptide interactions for use in wound healing applications.

## 1. Introduction

Circular dichroism (CD) spectroscopy is the most widespread technique used for estimating the secondary structures of proteins and polypeptides in solution [1]. This technique can be used to distinguish between unordered (random coil) and ordered (alpha-helix or beta-sheet) structures [2,3]. CD detects wavelength-dependent differences in the absorption of right and left circularly polarized light by optically active molecules such as peptides and proteins. The CD spectrum of unordered peptides is usually characterized by a single band below 200 nm, whereas alpha-helical structures usually present two negative bands at 208 and 222 nm along with one positive band at 192 nm; beta-sheet structures typically show a negative band at 217 nm and a positive band at 195 nm. Most linear cationic antimicrobial peptides (AMPs) are in an unordered state in aqueous solution. As these molecules are amphipathic, they can adopt folded conformations in both hydrophobic and hydrophilic environments [4]. AMPs are generally variable in length, sequence, and structure (helical, beta-sheet, extended, and looped) [4–6]. Alpha-helical AMPs are one of the most abundant types of AMPs. CD methods are very useful for studying protein-ligand interactions and protein denaturation due to their quantitative nature. Thus, any change in the CD spectrum of a protein upon addition of ligand, denaturant, or heat is directly proportional to the amount of protein perturbed [7].

In this study, we synthesized (KW)_4_ peptide based on previous data showing that (KW)_3_ has antimicrobial activity [8]. Another peptide with antimicrobial activity, HPA3NT3-analog, was also used [9]. Furthermore, we used three natural peptides, NRC-16, magainin-II, and reduced glutathione (GSH), in this study. NRC-16 peptide is derived from flatfish genes [10] and shows potent antimicrobial activity. Magainin-II was originally isolated from the skin of African clawed frog, *Xenopus laevis* [11]. GSH is a water-soluble tripeptide composed of the amino acids cysteine, glutamic acid, and glycine. The biological (antimicrobial or antioxidative) activities of these peptides promote interactions with gelatin, an important connective tissue protein. Here, we used CD spectroscopy to monitor the gelatin response to these peptides, sodium dodecyl sulfate (SDS), or calcium chloride (CaCl_2_). The structures and organization of the peptides in aqueous solution, SDS, Tween 80, lipopolysaccharide (LPS), mannan, or laminarin were determined using CD spectroscopy. The peptide-dependent interactions in gelatin, SDS, LPS, mannan, and laminarin were investigated by tryptophan (Trp) blue shift assay. Finally, the antibacterial and cytotoxicity activities of the peptides were determined.

## 2. Results and Discussion

Gelatin is a protein produced by acid and alkaline processing of collagen and is characterized by a three-chain structure in which individual helical chains are stranded in a superhelix about a common molecular axis [12–14]. The triple helical structure of gelatin can be quantified by using CD measurements [15]. In the present study, the CD spectra of gelatin showed two peaks, a negative peak at 205 nm suggesting a random coil conformation, and a positive peak at 222 nm characteristic of the triple-helial conformation of gelatin [16–19]. This positive peak corresponds to the typical maximum peak of collagen at 222 nm [20–27]. The triple helical structure of collagen was established by Ramachandran and Kartha [28], Rich and Crick [29], and Cowan *et al.* [30].

### 2.1. Effects of Antimicrobial Peptides on Gelatin Conformation

**Figure 1:**
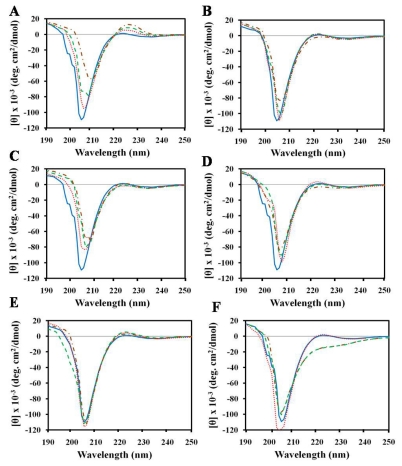
Effects of peptides on Circular dichroism (CD) spectra of gelatin in the wavelength region from 250–190 nm. Gelatin solution was treated with peptides at an increasing molar ratio of gelatin to peptide from 11:1 to 11:3 and incubated for 10 min at 25 °C. (**A**) (KW)_4_; (**B**) HPA3NT3-analog; (**C**) NRC-16; (**D**) Magainin-II; and (**E**) Reduced glutathione (GSH). CD spectra of 0.1% gelatin in the absence of peptides (solid line). Peptide concentrations were as follows: 50 μM (dotted line), 100 μM (dashed line), and 200 μM (dashed-dotted line). (**F**) CD spectra of gelatin in the absence (solid line) or presence of 1 mM SDS (dotted line), 5 mM SDS (dashed line), and 10 mM SDS (dashed dotted line).

CD spectroscopy has been used to characterize the interactions between small molecules and collagen with the aim of determining collagen stability [24,25,27,31–33]. We have also characterized the effects of peptides on the structural conformation of gelatin for biomedical applications. We prefer gelatin over collagen since the cost of producing collagen-containing sheets is higher than that of gelatin-containing sheets. Additionally, gelatin is water-soluble compared to the acid and salt solubilility of collagen, which is very important in peptide-based drug development. The CD spectra of gelatin solutions treated with various concentrations of peptides at 25 °C are shown in Figure 1.

Compared to gelatin solution alone, the gelatin-peptide mixture displayed a marked decrease in the negative intensity of the dichroic spectrum as well as a slight decrease or increase in the molar ellipticity of the 222 nm bands. In addition, the negative peak shifted to a higher wavelength with increasing concentration of peptides. It has been reported that upon complete denaturation of gelatin, the positive peak at 222 nm disappears completely while the negative band shifts to nearly 230 nm [18]. However, our results showed that the addition of peptides to the gelatin solution did not cause the positive band at 222 nm to disappear, and there was a slight change in the negative band for the gelatin-peptide complex. This shows that peptide brought about a very slight change in the packing of the helices and did not change the triple helical conformation of gelatin. It was reported earlier that the interaction of collagen with glycoprotein [34] or small molecules such as polyphenols [24], curcumin [25], dicarboxylic acids [27], chromium [21], tannins [35], aldehydes [36], and 3,4-dihydroxyphenylalanine [37] does not alter the secondary structural conformation of collagen. Other studies reported that the triple helicity of collagen is not affected by AMP (pexiganan, an analog of magainin [38]) incorporation as confirmed by FT-IR [39]. Maintaining the triple helical conformation of gelatin or collagen-based biomaterials during preparation is important in eliciting the desired biomedical functions of both.

The CD spectra of gelatin with peptides displayed a decrease in negative ellipticity, suggesting that the peptides bound intra-molecularly, *i.e.*, within a gelatin molecule, or promoted aggregation of gelatin. This point is likely correlated with aggregation of collagen-glycoprotein [34] or small molecule interactions [24,27]. On the other hand, the result that GSH did not increase negative ellipticity indicates that the gelatin molecules did not aggregate in the presence of GSH peptide. Thus, peptide promoted the native state of gelatin without affecting the peaks at 222 nm, as confirmed by CD measurements. It has been suggested that the presence of GSH in the gelatin matrix may have antioxidant effects in a wound environment such as, preventing damage to important cellular components caused by reactive oxygen species such as free radicals and peroxides [40]. Moreover, the antioxidant function of biotinylated matrikine peptide has been shown to enhance wound healing in rats [41].

### 2.2. Effect of SDS on Gelatin Conformation

SDS is an anionic additive frequently used in studies on protein denaturation, and it is a well-known destabilizing agent of biopolymers. To confirm that the peptide does not alter the triple helical content of gelatin, we used SDS to denature gelatin. This procedure can be used to differentiate proteins with a triple helical conformation from others with a non-triple helical conformation [18]. Figure 1F shows the CD spectra of a 0.1% gelatin solution in various concentrations of SDS at 25 °C. The triple helical content was reduced with increasing concentration of SDS. At increasing surfactant concentrations, absence of the 222 nm peak was observed. A previous study also reported that SDS eliminates the 222 nm peak [18]. Denaturation also occurred in 3 M CaCl_2_ solution, as indicated by the disappearance of the 222 nm peak (data not shown).

### 2.3. Structures of Peptides in Aqueous and SDS Solution

**Figure 2:**
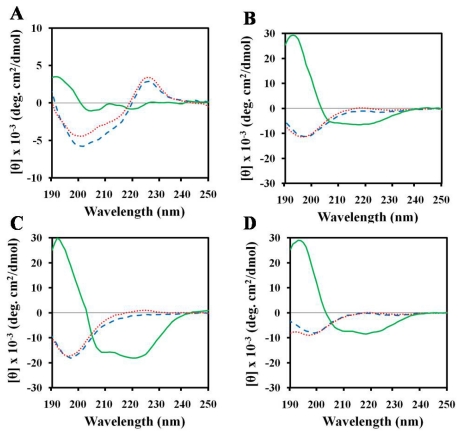
CD spectra of peptides in aqueous solution (dashed line) as well as in the presence of 0.1% Tween 80 (dotted line) and 30 mM sodium dodecyl sulfate (SDS) (solid line). (**A**) (KW)_4_; (**B**) HPA3NT3-analog; (**C**) NRC-16; and (**D**) Magainin-II.

In order to examine the behavior of the AMPs in membrane-mimicking media, the CD spectra of the peptides were recorded in the absence and presence of 30 mM SDS (Figure 2). The peptides showed a random coil conformation in aqueous solution. The conformations of the three peptides magainin-II, HPA3NT3-analog, and NRC-16 in SDS micelles included significant alpha-helical content. The alpha-helical contents of HPA3NT3-analog, magainin-II, and NRC-16 were 19, 29, and 59%, respectively. In the presence of SDS micelles, (KW)_4_ peptide exhibited a slightly folded conformation in which Trp residues were positioned mostly near the membrane-water interfacial region [42], suggesting interfacial association. Such a structural feature is believed to be important for the antibacterial activity of linear AMPs [43–46].

### 2.4. Structures of Peptides in Nonionic Surfactant

Tween 80 belongs to a class of nonionic surfactants that are frequently used in membrane protein solubilization, since they generally do not induce protein folding. Its chemical stability and high biocompatibility mean that it can be regarded as a strong candidate for future use in cosmetics and drug delivery. Based on CD, we investigated whether or not Tween 80 causes changes in peptide conformation. To accomplish this, we determined the conformations of peptides in the presence of Tween 80 (Figure 2). According to the results, Tween 80 did not alter peptide conformation. In addition, Trp containing (KW)_4_ peptide showed a negative band at 200 nm. This band is characteristic of a random coil, whereas the band at 225 nm was related to the Trp side chain in (KW)_4_, which contributes to the CD signal in this spectral region [47–49]. On the other hand, the Trp side chain peak did not disappear in response to Tween 80, indicating that there was no hydrophobic interaction between the peptide and Tween 80. This is further supported by a previous study that did not find any general structural changes in insulin in the presence of Tween 80, which suggests that only limited interactions, if any, occur between the two species in solution [50]. Clearly, this result shows that Tween 80 had no harmful effects on the peptides, which could be useful for the cosmetic application of these peptides using Tween 80 as a co-surfactant.

### 2.5. Structures of Peptides in Cell Wall Components

**Figure 3:**
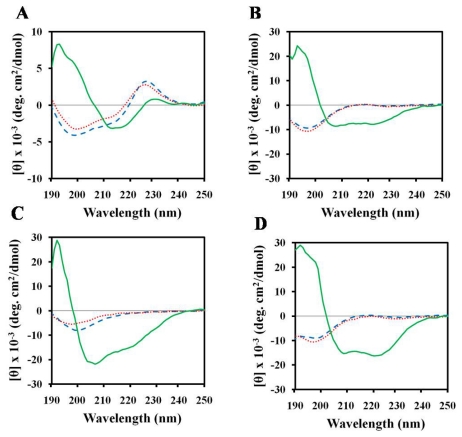
CD spectra of peptides in the presence of 0.1% mannan (dashed line), 0.1% laminarin (dotted line), and 0.1% lipopolysaccharide (LPS) (solid line). (**A**) (KW)_4_; (**B**) HPA3NT3-analog; (**C**) NRC-16; and (**D**) Magainin-II.

Finally, we used CD to investigate the conformational changes brought on by the interactions between peptides and cell wall components (LPS, mannan, and laminarin). CD spectroscopy showed that (KW)_4_ peptide displayed major conformational changes in association with LPS (Figure 3), which is a cell wall component of Gram-negative bacteria [51].

On the other hand, the peptides magainin-II, NRC-16, and HPA3NT3-analog transitioned from a random coil conformation to an alpha-helix upon interaction with LPS. Examples of other native, synthetic peptides that undergo alpha-helix formation upon LPS binding include melittin and magainin [52,53]. The ability of AMPs to bind LPS is a prerequisite for their antibacterial and endotoxin detoxifying activities [53]. Additionally, we determined whether or not binding of these peptides to fungal surfaces occurs via interaction with mannan or laminarin, which are major components of fungal cell walls. CD spectroscopy showed that these peptides displayed no major conformational changes associated with the fungal cell wall components mannan and laminarin (Figure 3). Therefore, it is clear that cell wall components did not affect the fungicidal activities of these peptides. This point suggests that the antifungal activity of AMPs is not affected by the removal of cell wall components [54,55].

### 2.6. Characterization of the Trp Environment Using Fluorescence Spectroscopy

The peptide-binding process can also be followed by analysis of the Trp flourescence spectra (Table 1).

The (KW)_4_ and NRC-16 peptides were used as they contain Trp residues in their sequences. In the presence of sodium phosphate (SP) buffer (pH 7.2), (KW)_4_ and NRC-16 peptides displayed fluorescence emission maxima at 355 and 356 nm, respectively, which corroborates a previous report that the Trp residues of these peptides are located in a hydrophilic environment [56]. When these two peptides bound to gelatin, SDS micelles, and LPS, their fluorescence maxima shifted to shorter wavelengths, suggesting that the Trp side chains partitioned preferentially into more rigid, hydrophobic environments in gelatin, SDS, and LPS. This tendency is consistent with the CD spectra of these peptides in the presence of SDS micelles or LPS, which indicated a much more structured conformation upon binding (Figures 2,3). The recorded blue shift suggests that Trp residues were involved in the interaction with the cationic or hydrophobic domain of gelatin. Cationic peptides are thought to undergo electrostatic, cation-π, and hydrophobic interactions with gelatin or collagen. Gelatin consists of positively and negatively charged as well as hydrophobic domains. A previous study reported that hydrophobic interactions are mainly involved in the interactions between polyphenols and collagen [24]. Bioinformatics studies have also shown that quinone components interact with collagen through hydrophobic interactions [57]. Lysine (Lys) and arginine (Arg) residues mediate electrostatic interactions that attach cationic peptides to negatively charged amino acids in gelatin molecules. It has been suggested that cation-π interactions between a protonated amine (Lys) or guanidine (Arg) side chain and an aromatic ring side-chain (phenylalanine, tyrosine, or Trp) promote peptide-gelatin interactions [58,59]. Therefore, electrostatic, hydrophobic, and cation-π interactions might contribute to peptide-gelatin interactions.

### 2.7. Antibacterial and Cytotoxicity Activities of Peptides

A high level of microorganisms inhibits the normal wound healing process [60]. Wound infections are most frequently attributed to *P. aeruginosa* [61]. The MIC values of the peptides against *P. aeruginosa* under low salt (without NaCl) and high salt (with 135 mM NaCl) conditions are summarized in Table 2.

**Figure 4:**
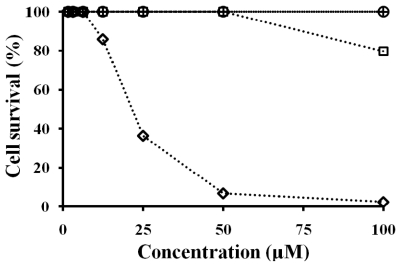
Activities of the peptides against HaCaT cells. HaCaT cells (2 × 10^4^/well) were incubated for 24 h with the indicated concentrations of (KW)_4_ (+), HPA3NT3-analog (⋄), NRC-16 (□), and magainin-II (○).

Antibacterial activity was measured in 10 mM sodium phosphate buffer, pH 7.2, and phosphate buffered saline, pH 7.2 (number in the parentheses). NRC-16 peptide showed higher antibacterial activity than the other peptides. However, all of the peptides showed antibacterial activity against *P. aeruginosa* under both low and high salt conditions. The cytotoxic activities of the four peptides were assessed in HaCaT cells as a measurement of their toxicity towards higher order eukaryotic skin cells (Figure 4). At a concentration of 100 μM, (KW)_4_ and magainin-II peptides displayed non-cytotoxicity towards HaCaT cells, whereas NRC-16 and HPA3NT3-analog peptides showed cytotoxicity. Therefore, the (KW)_4_ and magainin-II peptides were selective against bacteria with no effect on HaCaT cells. The ability to localize AMPs to wound sites is important for the effective treatment of bacterial infection at wound sites. Previous studies provide rationale for the application of collagen membranes to AMP delivery in infected wounds [39,41,62]. Other studies have reported that gelatin sheets containing bioactive molecules possess effective wound healing activity [63–66]. Gelatin gel shows non-cytotoxicty towards HaCaT cells [67] and is biodegradable in nature [68]. Additionally, it has good film forming properties and can be used in wound healing by preventing fluid loss due to exudation [69]. Gelatin gel is also effective in peptide delivery and has antibacterial effects at wound sites. Cationic peptides may also possess anticancer activity [70,71] or promote wound healing [72], and several AMPs are currently undergoing clinical trials [73]. Therefore, gelatin-peptides sheets could be useful for the inhibition of microbes at wound sites due to their wound healing properties.

## 3. Experimental Section

### 3.1. Materials

Rink amide 4-methylbenzhydrylamine resin, fluoren-9-ylmethoxycarbonyl (Fmoc) amino acids, and other reagents for peptide synthesis were purchased from Calibochem-Novabiochem (La Jolla, CA, USA). LPS from *P. aeruginosa*, GSH, and Tween-80 were purchased from Sigma Chemical Co. (St. Louis, MO, USA). Granulated gelatin was obtained from DIFCO Laboratories (Detroit, MI, USA). The anionic surfactant, SDS, and CaCl_2_ were acquired from Calbiochem (La Jolla, CA, USA). All other reagents were of analytical grade. Buffers were prepared using double distilled water (Millipore Co.).

### 3.2. Peptide Synthesis and Purification

The peptides KWKWKWKW-NH_2_ (KW)_4_, FKKLKKLFKKILKLK-NH_2_ (HPA3NT3-analog), GWKKWLRKGAKHLGQAAIK-NH_2_ (NRC-16), and NMIEGVFAKGFKKASHLFKGIG (magainin-II) were synthesized by the solid-phase method using Fmoc chemistry on a solid support of Rink amide 4-methylbenzhydrydrylamine resin. Then, 0.1 M *N*-hydroxy benzotriazole (HOBt) and 0.45 M 2-(1*H*benzotriazole- 1-yil)-1,1,3,3-tetramethyluroniumhexafluorophosphate (HBTU) in dimethylformamide (DMF) along with 2 M *N*,*N*-diisopropyl ethylamine (DIEA) in *N*-methylpyrrolidone (NMP) were used as a coupling reagent, and 10-fold excess Fmoc-amino acid was added during every coupling cycle. Following a final deprotection with a solution of 20% piperidine in DMF and cleavage with a mixture of TFA/water/triisopropylsilane (90:5:5) for 2 h at room temperature [74], the crude peptides were repeatedly extracted with diethyl ether and purified using reverse phase preparative HPLC on a Vydac C_18_ column (4.6 × 250 mm, 300 Å, 5 nm). The molecular masses of the peptides were confirmed by using a matrix-assisted laser desorption ionization mass spectrometer (data not shown) (MALDI II, Kratos Analytical Ins.).

### 3.3. Interaction of Peptides with Gelatin by CD Spectroscopy

Gelatin solutions were prepared by weighing the required amount of gelatin flakes and soaking in hot water (~40 °C) with stirring. The concentrations quoted here are expressed in weight percentage of gelatin.

CD spectra were recorded at 25 °C on a Jasco 810 spectropolarimeter (Jasco, Tokyo, Japan) equipped with a temperature control unit using a 0.1-cm path-length quartz cell. The gelatin (0.1%) was scanned in the presence of 10 mM SP buffer. The CD spectra were measured for the 0.1% gelatin samples (dissolved in 10 mM SP buffer (pH 7.2)) containing peptide. CD data represent the average value of three separate recordings with four scans per sample. A reference spectrum containing 10 mM SP buffer was also recorded. The CD spectra of the samples were obtained after subtracting the reference spectrum. Changes in the conformation of gelatin upon addition of peptides were recorded.

### 3.4. Interaction of Peptides with Surfactants by CD Spectroscopy

The CD spectra of the peptides (50 μM) were obtained in different environments, including 10 mM SP buffer, 30 mM SDS, and 0.1% Tween 80. Ten millimoles of SP buffer was used to prepare 30 mM SDS and 0.1% Tween 80. At least five scans in the 250–190 nm wavelength range were conducted, and the average blank spectra were subtracted from the average of the sample spectra. All CD spectra are presented as the mean residue ellipticity, [*θ*]_MRW_, in deg·cm^2^/dmol. The alpha-helical content was determined from the mean residue ellipticities at 222 nm, as indicated in Equation 1 [75]:

(1) $$\%\text{Helix}=({\left[\theta \right]}_{\text{obs}}\times 100)/\{{\left[\theta \right]}_{\text{helix}}\times (1-2.57/l)\}$$

where [*θ*]_obs_ is the mean-residue ellipticity observed experimentally at 222 nm, [*θ*]_helix_ is the ellipticity of a peptide of infinite length with a 100% helix population, taken as −39,500 deg·cm^2^/dmol, and *l* is the peptide length or, more precisely, the number of peptide bonds.

### 3.5. Interaction of Peptides with Cell Wall Components by CD Spectroscopy

The peptides were scanned in the presence or absence of LPS (0.1%) dissolved in 10 mM SP buffer. The secondary structures were monitored at a peptide concentration of 50 μM in 10 mM SP buffer in the presence of laminarin from digitata laminarin (0.1%; Sigma-Aldrich, St. Louis, MO, USA) and in the presence of mannan from *Saccharomyces cerevisiae* (0.1%; Sigma-Aldrich, St. Louis, MO, USA). CD data represent the average value of three separate recordings.

### 3.6. Trp Fluorescence Assay

The fluorescence emission spectra of the Trp residues in the peptides were monitored in the presence of 10 mM SP buffer, 0.1% gelatin, 30 mM SDS, cell wall components (0.1% LPS, 0.1% laminarin, and 0.1% mannan), and 0.1% Tween 80. The Trp fluorescence measurements were taken using a spectrofluorometer. The final concentration (2 μM) of each peptide was added to 200 μL of the above solutions, and each peptide: environment mixture was allowed to interact at 25 °C for 10 min. Fluorescence was measured at an excitation wavelenth of 280 nm and an emission wavelength from 300 to 400 nm.

### 3.7. Antibacterial Activity

The antibacterial activities of the peptides against drug-resistant *P. aeruginosa* (3547, 3592, 4007, 4891) were examined using the microbroth dilution method. Aliquots of bacterial suspensions (50 μL) in mid-log phase at a concentration of 2 × 10^5^ colony forming units (CFUs/mL) in Mueller Hinton Broth (MHB, BD, Sparks, MD, USA) culture medium were added to each well containing 50 μL of the peptide solution that had been serially diluted 2-fold in buffer (10 mM SP buffer, pH 7.2 or phosphate buffered saline (PBS); 1.5 mM KH_2_PO_4_, 2.7 mM KCl, 8.1 mM Na_2_HPO_4_, 135 mM NaCl, pH 7.2). Several wells were kept untreated as a control in order to monitor bacterial growth. Inhibition of bacterial growth was determined by measuring the absorbance at 620 nm using a Versa-Max microplate Elisa Reader (Molecular Devices, Sunnyvale, CA, USA) after incubation for 18 h at 37 °C. The MIC is defined as the minimal peptide concentration that inhibits bacterial growth. All MIC measurements are the average of three to four independent experiments. The bacterial strains were procured from Chonnam University Hospital, in Gwangju, South Korea. All isolates were stored at −70 °C until required.

### 3.8. Cell Culture and Cytotoxicity

To examine the cytotoxic effects of the peptides, HaCaT (human keratinocyte cell line) cells were cultured in Dulbecco’s modified Eagle medium (DMEM) supplemented with antibiotics (100 U/mL of penicillin, 100 μg/mL of streptomycin) and 10% fetal calf serum at 37 °C in a humidified chamber containing 5% CO_2_. The percentage of growth inhibition was evaluated by MTT (Sigma) assay for the measurement of viable cells. A total of 2 × 10^4^ cells/well was seeded onto a 96-well plate and then incubated for 24 h. Various concentrations of the test peptides were then added to the wells, after which the cells were incubated for an additional 24 h at 37 °C. Subsequently, 10 μL of MTT at a concentration of 5 mg/mL was added to each of the wells, after which the cells were incubated for an additional 4 h. The supernatants were then aspirated, and 100 μL of dimethyl sulfoxide was added to the wells in order to dissolve any remaining precipitate. Absorbance was then measured at a wavelength of 570 nm using an EL_x_800 reader (Bio-Tek instruments, Inc., Winooski, VT).

## 4. Conclusion

The native structure of gelatin was not altered upon treatment with peptides. Therefore, changes in the structural properties of gelatin upon interaction with peptides assist the preparation of gelatin-peptide sheets. In this study, the tested peptides showed antimicrobial activity, which suggests that they might be useful in the development of a topical application for wound sites. Among them, (KW)_4_ possesses a simple composition with microbial selective properties, making it economically viable for many applications, including inhibition of bacterial infection at wound sites.

## Figures and Tables

**Table 1:** Tryptophan (Trp) emission maxima of 2 μM peptides in 10 mM sodium phosphate buffer (pH 7.2), gelatin, sodium dodecyl sulfate (SDS), Tween 80, mannan, laminarin, and lipopolysaccharide (LPS).

Blue shift (nm)							

Peptides	λ_max_ buffer (nm)	Gelatin	SDS	Tween	Mannan	Laminarin	LPS
(KW)_4_	355	8	10	0	0	0	10
NRC-16	356	8	25	0	0	0	17

**Table 2:** MICs of the peptides against drug–resistant *P. aeruginosa*.

MIC (μM)				

Resistant strains	(KW)_4_	HPA3NT3-analog	NRC-16	Magainin-II
*P. aeruginosa* 3547	16 (32)	8 (16)	4 (8)	32 (64)
*P. aeruginosa* 3592	32 (64)	8 (16)	8 (16)	32 (64)
*P. aeruginosa* 4007	16 (32)	4 (16)	4 (8)	32 (32)
*P. aeruginosa* 4891	8 (16)	4 (8)	4 (8)	16 (32)
